# Securing Industrial Control Systems: Components, Cyber Threats, and Machine Learning-Driven Defense Strategies

**DOI:** 10.3390/s23218840

**Published:** 2023-10-30

**Authors:** Mary Nankya, Robin Chataut, Robert Akl

**Affiliations:** 1 Computer Science Department, Fitchburg State University, Fitchburg, MA 01420, USA; 2 School of Computing and Engineering, Quinnipiac University, Hamden, CT 06514, USA; robin.chataut@quinnipiac.edu; 3 Department of Computer Science and Engineering, University of North Texas, Denton, TX 76203, USA; robert.akl@unt.edu

**Keywords:** cyber defense, industrial control systems, SCADA, anomaly detection, cyber threats, vulnerabilities, attacks, artificial intelligence, security

## Abstract

Industrial Control Systems (ICS), which include Supervisory Control and Data Acquisition (SCADA) systems, Distributed Control Systems (DCS), and Programmable Logic Controllers (PLC), play a crucial role in managing and regulating industrial processes. However, ensuring the security of these systems is of utmost importance due to the potentially severe consequences of cyber attacks. This article presents an overview of ICS security, covering its components, protocols, industrial applications, and performance aspects. It also highlights the typical threats and vulnerabilities faced by these systems. Moreover, the article identifies key factors that influence the design decisions concerning control, communication, reliability, and redundancy properties of ICS, as these are critical in determining the security needs of the system. The article outlines existing security countermeasures, including network segmentation, access control, patch management, and security monitoring. Furthermore, the article explores the integration of machine learning techniques to enhance the cybersecurity of ICS. Machine learning offers several advantages, such as anomaly detection, threat intelligence analysis, and predictive maintenance. However, combining machine learning with other security measures is essential to establish a comprehensive defense strategy for ICS. The article also addresses the challenges associated with existing measures and provides recommendations for improving ICS security. This paper becomes a valuable reference for researchers aiming to make meaningful contributions within the constantly evolving ICS domain by providing an in-depth examination of the present state, challenges, and potential future advancements.

## 1. Introduction

Industrial Control System (ICS) is an encompassing term that refers to various control systems and their associated instrumentation. It encompasses a diverse array of equipment, systems, networks, and mechanisms employed for the purpose of managing and automating industrial operations [1]. Virtually every commercial building and industrial facility, including those in production, transportation, power generation, and water treatment, relies on ICS devices and protocols. These systems heavily depend on the automation of mechanical and electrical processes. However, their connectivity to the internet poses a significant vulnerability, making them susceptible to cyber-attacks [2]. The global ICS market is experiencing substantial growth, primarily driven by the rising emphasis on automation, cloud computing, and digitization across various industries [3]. More innovative technologies are being developed, enabling remote access and control over the internet and within Information Technology environments. This shift towards increased automation and connectivity aims to achieve substantial business benefits. However, it also presents a challenge, as integrating Industrial Control Systems with external networks, such as the internet, expands the attack surface, making them more susceptible to cyber threats without proper security measures [4]. Over the past decade, cyber attacks on Industrial Control Systems have notably increased due to their heightened vulnerability to off-site attacks. Previously, these systems operated in isolated environments, relying heavily on human intervention. However, the growing inter-connectivity has exposed them to potential risks from remote adversaries. Consequently, ensuring robust security measures has become paramount to safeguarding ICSs from cyber threats [5].

**Figure 1:**
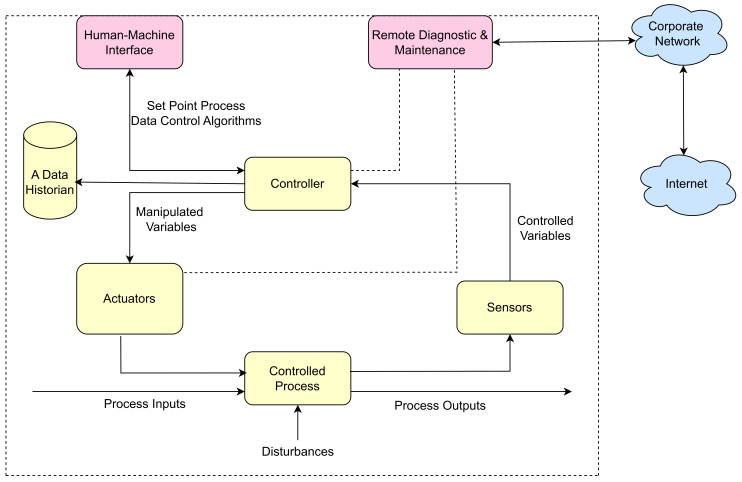
The basic components and operation of an Industrial Control System.

An overview of an ICS contains several control loops, remote diagnostics, maintenance tools, and human interfaces built on layered network architectures using various network protocols. A summary of the basic components and process of an ICS is shown in Figure 1.

- A process consists of activities to achieve the desired output;
- A control loop utilizes sensors, actuators, and controllers or PLCs to adjust the output value to the desired set-point automatically;
- A sensor detects changes in its environment and sends information as controlled variables to the controller;
- A controller uses target set point and control algorithms to generate required output variables and transmit them to the actuators;
- Actuators or movers are elements within a machine designed for the manipulation or regulation of a mechanism, such as control valves, circuit breakers, switches, and motors;
- Human–Machine Interfaces (HMI) are utilities used to display process status information and monitor and configure controller parameters;
- Remote Diagnostics and Maintenance applications do real-time diagnosis and maintenance operations such as remotely identifying, preventing, and recovering from abnormal operations or failures;
- A Data Historian is a centralized database storing all process information within an ICS environment. The logged data is exported to the corporate Information Systems (IS) for process data analysis, control, and planning;
- A Communications Gateway device enables communication with a distant network, such as the internet or an autonomous system, which is not accessible to the host network nodes. This gateway can be realized using either hardware or software. It directs the network traffic and may block specific traffic to protect it from malicious attacks. It also grants or denies access to computers within the network to the outside world.

In the forthcoming part of this article, Section 2 provides a more in-depth review of the ICS technologies. Section 3 outlines the system design considerations that help to determine the security needs of the ICS system. In Section 4, we present some popular examples of ICS cyber attack incidents that have occurred in the past. Section 5 discusses the vulnerability of ICS. Section 6 offers an in-depth review of ICS communication protocols for cyber vulnerabilities. We further delve into a comprehensive review of the existing measures to strengthen the cybersecurity of ICSs in Section 7. In Section 8, we explore how machine learning has been integrated to strengthen the cyber defense for ICS. In Section 9, we discuss the challenges of machine learning approaches and mechanisms for defending ICS. Section 10 presents a list of recommendations and the future direction of our research. Finally, we conclude our study in Section 11 where we summarize the key fundamental research explorations, emphasizing the future direction of our work.

## 2. Industrial Control Systems Technologies

ICS has different technologies such as SCADA, DCS, Industrial Automation and Control Systems (IACS), PLCs, Programmable Automation Controllers (PACs), HMI, RTUs, control servers, Intelligent Electronic Devices (IEDs), and sensors [6]. The integration of these features contributes to the widespread adoption of Industrial Control Systems, leading to a market value of USD 130,060 million in 2022. The market is expected to experience a Compound Annual Growth Rate (CAGR) of 7.55% from 2023 to 2030, primarily driven by the increasing demand for energy-efficient and safe operations [7].

### 2.1. Supervisory Control and Data Acquisition

SCADA is among the most widely utilized technologies in Industrial Control Systems [8]. It functions as a software application designed to control industrial processes by collecting real-time data from remote locations, allowing for the management of equipment and conditions [9]. SCADA systems are composed of both hardware and software components. The hardware gathers and sends data to field controller systems, which subsequently transmit the data to other systems for real-time processing and display through a HMI. Additionally, SCADA systems maintain a comprehensive record of all events, enabling the reporting of process status and any encountered issues. These applications also include alarm functions that notify operators when hazardous conditions arise, ensuring prompt and appropriate responses [10]. SCADA provides organizations with the tools to make and deploy data-driven decisions regarding their industrial processes [11]. Applications of SCADA include the below [12]:

- Electricity generation, transmission, and distribution;
- Manufacturing industries or plants;
- Food and pharma productions
- Telecom and IT-based systems;
- Traffic control;
- Lift and elevator control;
- Oil and gas systems;
- Mass transit and railway traction.

**Figure 2:**
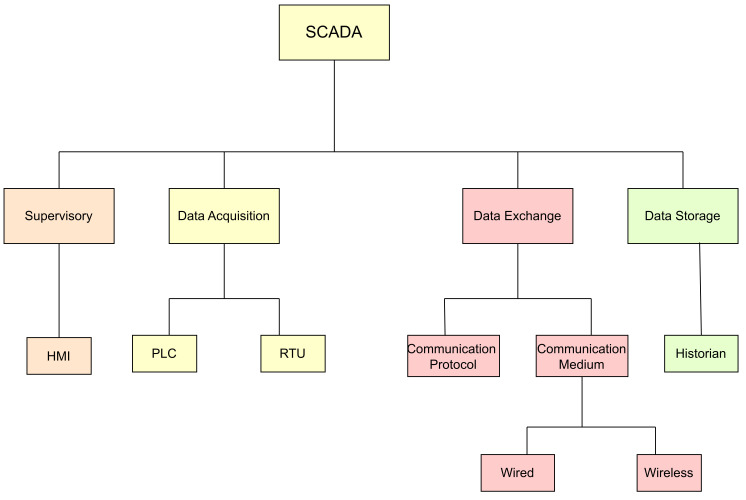
SCADA components.

SCADA employs a central computer to store information related to local and remote devices, enabling the control of industrial processes and facilities. The typical components of SCADA can be classified based on their respective definitions, as depicted in Figure 2 below.

- Supervisory control: Supervisory control serves as the fundamental role of the HMI. HMI software serves as an interface responsible for overseeing industrial processes. On the other hand, a master terminal unit (MTU) functions as a central supervisory controller that communicates with lower field devices, such as RTUs, through the ICS network;
- Data acquisition: Data can be acquired from two primary sources in the context of SCADA system as PLCs and Remote Telemetry Units (RTUs). Both the PLCs and RTUs interface directly with actuators and sensors in the field. RTUs are specifically designed to interface with sensors and collect telemetry data, which they then transmit to a primary system for further action. On the other hand, PLCs interface with the actuators to maintain and control industrial processes based on the telemetry data collected by the RTUs [13]. PLCs and RTUs act as physical interfaces between SCADA systems and field devices. However, their communication with the SCADA system differs. RTUs are well-suited for wide geographical areas due to their use of wireless communication methods. In contrast, PLCs are more tailored to local control applications [14];
- Data storage: The majority of SCADA systems employ a Structured Query Language (SQL) database for storing data with timestamps. A historian is a fully integrated SCADA software that collects real-time data from various SCADA devices and stores them in a database, such as mySQL;
- Data exchange: Communication protocols are used to exchange data between SCADA components.

#### SCADA Architecture

This section describes the four generations of SCADA architecture in detail and summarizes the security strengths and vulnerabilities of each.

**Figure 3:**
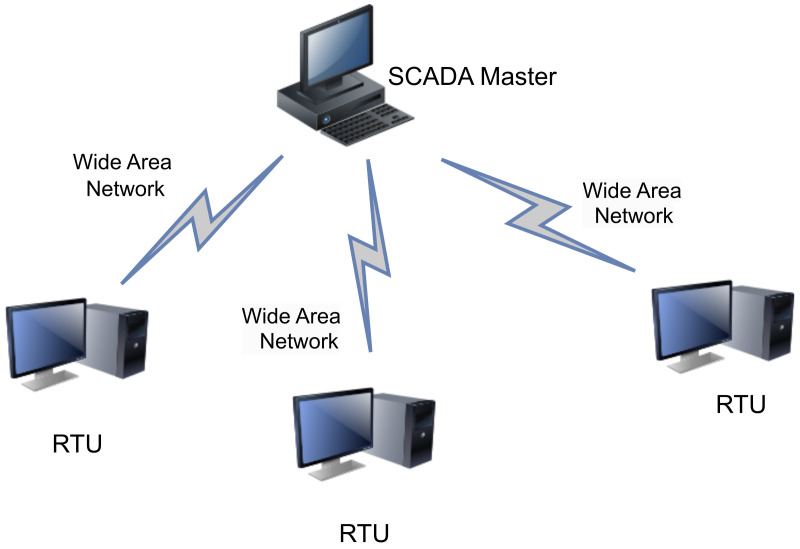
Monolithic SCADA system.

**Figure 4:**
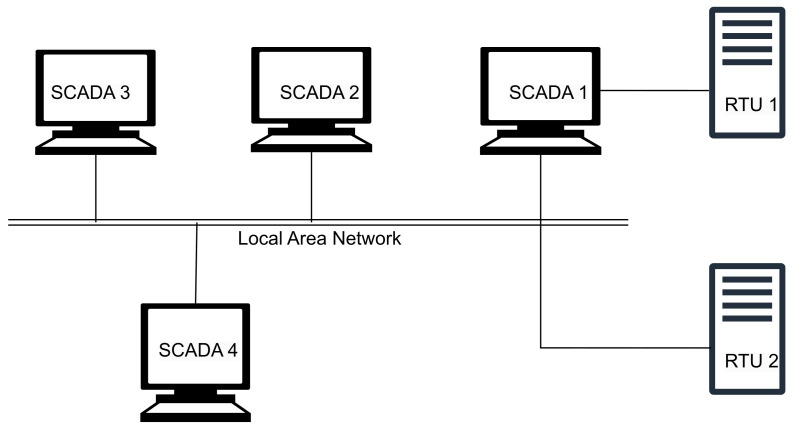
Distributed SCADA system.

**Figure 5:**
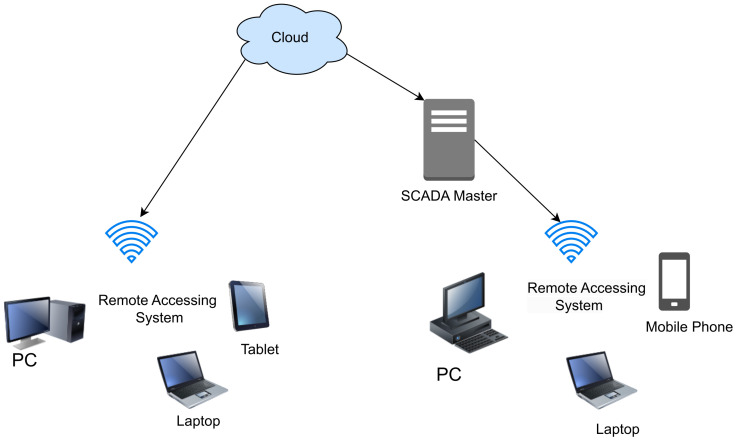
Internet of Things (IoT) SCADA system.

**Figure 6:**
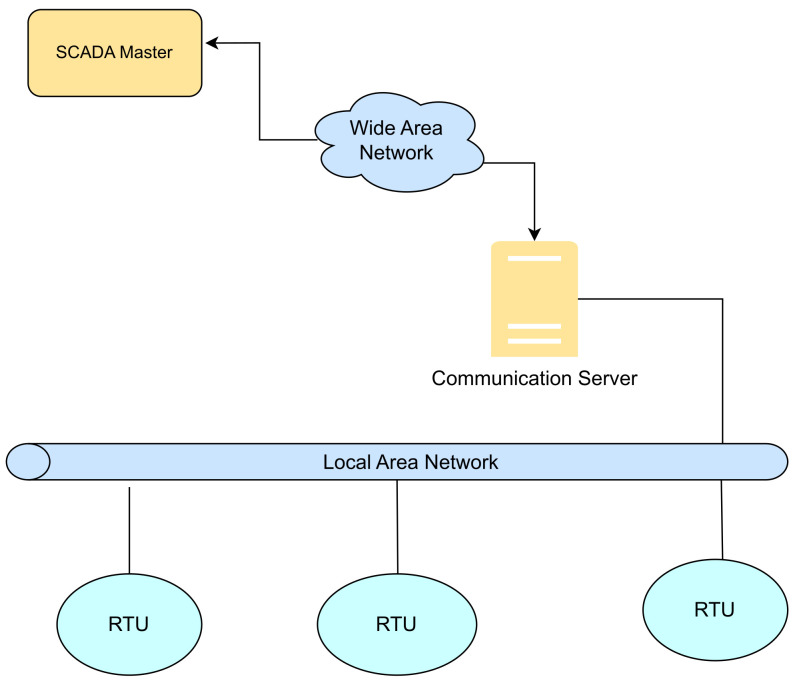
Networked SCADA architecture.

(a) First generation-Monolithic: The first generation of SCADA systems was developed when networks were not yet in existence. These early systems were not designed to connect with other systems, and communication was typically limited to Wide Area Networks (WANs) interacting with remote terminal units (RTUs) [15]. It defines application in remote areas within a factory where the conditions are unsafe, and physical access is restricted [16]. In the early-generation systems, redundancy was achieved by deploying two mainframe systems with identical configurations. One was designated as the primary and the other as the backup. These two systems were connected at the bus level. The standby system’s main role was to act as a monitoring entity for the primary system and would smoothly take over if it detected any indications of failure. Consequently, the standby system usually operated in an idle state, performing minimal to no processing tasks until a fail-over event became necessary [17]. Figure 3 shows a typical first-generation SCADA architecture.
(b) Distributed SCADA system: Control functions were distributed across multiple systems during second generation [18]. Distributing the individual functions of the SCADA system across multiple systems resulted in a collective processing power that exceeded what could have been achieved with a single processor [19]. During the 1980s, SCADA systems harnessed the widespread adoption of proprietary local area networks(LAN) and more compact yet potent computers. This facilitated enhanced sharing of operational data not only within the plant but also at broader levels. These individual stations were used to share real-time information and command processing for performing control tasks to trip the alarm levels of possible problems. Only the developers cared about the SCADA security [20]. Figure 4 below shows the Distributed SCADA architecture [21].
(c) Internet of Things (IoT): IoT introduces a distinct approach to SCADA systems, substituting the requirement for PLCs with an emphasis on data modeling and advanced algorithms. This transition signifies a departure from the traditional reliance on mainframes or server in a facility, as data goes to cloud-based servers for sharing and storage [22]. IoT SCADA systems are flexible and easy to maintain and integrate. IoT brought several other advantages to SCADA, such as ease of use, flexibility, availability, cost efficiency, big data processing, and scalability [23]. Figure 5 below shows the IoT SCADA architecture.
(d) Networked SCADA Architecture: During the third generation, the monitoring process heavily relied on the involvement of PLCs. They were integrated into the SCADA system, providing efficient and reliable data acquisition and control capabilities. This integration of PLCs enhanced the overall functionality and responsiveness of the SCADA system, enabling real-time monitoring and control of industrial processes across a distributed network. The third-generation SCADA architecture thus facilitated greater flexibility, scalability, and accessibility, making it more adaptable to modern industrial demands [24]. It can connect to the internet and third-party peripherals. Additionally, this architecture enhanced the performance level of SCADA by allowing several servers to run in parallel to handle several tasks [25]. Figure 6 below shows the description of the Networked SCADA architecture.

### 2.2. Distributed Control Systems (DCS)

Distributed Control Systems are comprised of controllers, sensors, and actuators that are distributed across different spatial locations [26]. The entire system’s sub-components are controlled by multiple controllers, e.g., PLC [27]. DCS is frequently employed in various industrial process industries, including but not limited to the following:

- Agriculture;
- Chemical plants;
- Petrochemical and refineries;
- Nuclear power plants;
- Water and sewage treatment plants;
- Food processing;
- Automobile manufacturing;
- Pharmaceutical manufacturing.

**Figure 7:**
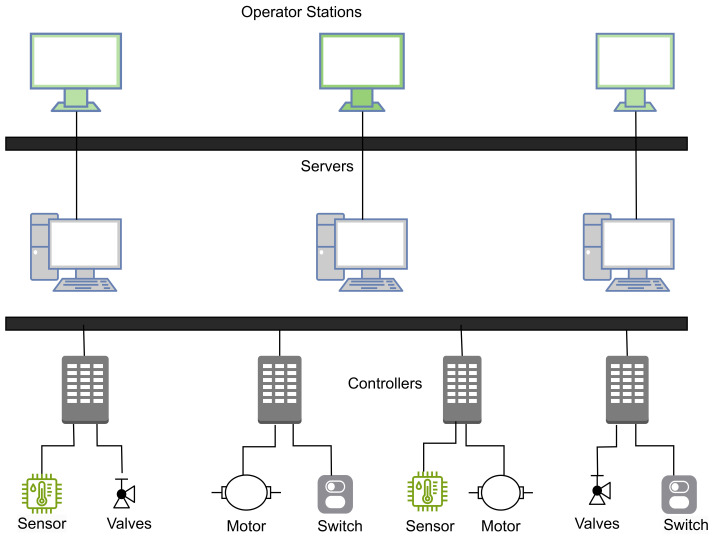
A distributed control system.

Within the domain of DCS, automatic control revolves around the exchange of signals, facilitating bidirectional information flow, and the computation of control actions through decision-making processes [28]. DCS is also defined as an architecture where the subsystems are geographically distributed and functionally integrated [29]. DCS coordinates and supervises a complete plant of many variable processes. See below a distributed control system in Figure 7.

#### 2.1.2. Function and Components of DCS

Components of DCS consist of the basic components, as listed below:

- An engineering workstation: This is the supervisory controller for the DCS as a whole. The station comes with configuration tools that empower users to undertake activities such as generating new loops, establishing input/output (I/O) points, and configuring distributed devices [30];
- An operator station: A station operator is a location where the user observes the ongoing process. At the station operator’s interface, the operator can access process variables, control parameters, and alarms, which are essential for retrieving the current operating status [31];
- A process control unit: This control center acts as the brain of all process control by performing all the computation process algorithms and running all logical expressions. The control module takes an input variable that will be controlled, calculates it, and the results are compared with the set point, which is the value expected of the process. If the calculation results differ from the set point, the value must be manipulated and the results sent to the actuator [31]. This controller, which relies on microprocessor technology, is specifically engineered for automatic and compound loop control;
- A communication system: This system facilitates the transfer of data from one station to another, a crucial function in distributed control systems. The network protocols employed encompass Ethernet, Profibus, and DeviceNet;
- Smart devices: These refer to intelligent devices or bus technologies employed to substitute older I/O systems.

### 2.3. Programmable Logic Controllers

PLCs are industrial computer control systems designed to constantly monitor the status of input devices and make decisions according to a customized program in order to manage the status of output devices [32]. Early PLCs were able to execute tens of instructions per second; modern PLCs can perform bit operations in nanoseconds. They can function as autonomous systems, optimizing processes intelligently and independently [33]. PLCs rely on a programmable memory that stores instructions for executing a wide range of operations, encompassing logic functions, sequence control, timing, counting, and arithmetic calculations. Using digital or analog input and output interfaces, this memory supervises and manages a variety of mechanical equipment and production processes [34]. Industries that rely on PLCs include the following:

- Oil and Gas;
- Food and Beverage;
- Automotive;
- Pharmaceuticals;
- Transportation;
- Off Road Construction;
- Lifts and escalators;
- Medical applications;
- Automatic gate systems;
- Heating control systems.

#### 2.3.1. Versions of PLCs

PLCs have evolved significantly, with a version incorporating Ethernet protocol based network connectivity that enables them to share data with a variety of devices and systems such as other PCs, SCADA, and even cloud-based platforms [35]. This enhanced connectivity and data sharing capability has further signified their pivotal role in ICS, as seen below.

- Real-Time Monitoring and Control: PLCs facilitate real-time monitoring and control of industrial processes. With their network connectivity, they can provide immediate data feedback, allowing for rapid decision-making and adjustments;
- Data Aggregation and Analysis: PLCs can collect and transmit data to centralized systems for analysis. This data is essential for process optimization, predictive maintenance, and quality control;
- Remote Accessibility: Connectivity enables remote accessibility to PLCs, allowing engineers and operators to manage and monitor processes from different locations, improving operational efficiency and reducing the need for onsite presence.

This version of the Ethernet protocol-based PLCs has several limitations despite its data sharing capability. These PLCs lacked standardization, leading to compatibility issues between devices from different manufacturers. They also present with data handling, processing, and storage limitations for more advanced applications. PLCs have become an integral part of the broader industrial landscape, especially within the frameworks of Industry 4.0 and the Industrial Internet of Things (IoT). A team of researchers proposed an IoT-PLC version that possesses regulatory control features, incorporates fog computing capabilities for tasks such as data filtering, field data storage, and supports various wireless interfaces that can be managed autonomously [36]. Their incorporation into these paradigms is of utmost importance and have solved the earlier mentioned limitations with the below capabilities, hence resulting into robust and secure solutions for modern industrial automation, as seen below [37].

- Enhanced Automation and Smart Manufacturing: PLCs contribute to the automation and intelligent control of industrial processes, aligning perfectly with the objectives of Industry 4.0 and industrial IoT, which aim to create smart and interconnected factories;
- Optimizing Resource Utilization: PLCs, as part of ICS, contribute to optimizing resource utilization, reducing energy consumption, and minimizing waste, which are central to sustainable and eco-friendly manufacturing practices;
- Data-Driven Decision Making: In Industry 4.0 and industrial IoT, data is a valuable asset. PLCs’ connectivity enables them to generate and share data, which is the foundation for data-driven decision-making, predictive maintenance, and process optimization.

#### 2.3.2. Components of PLC system

**Figure 8:**
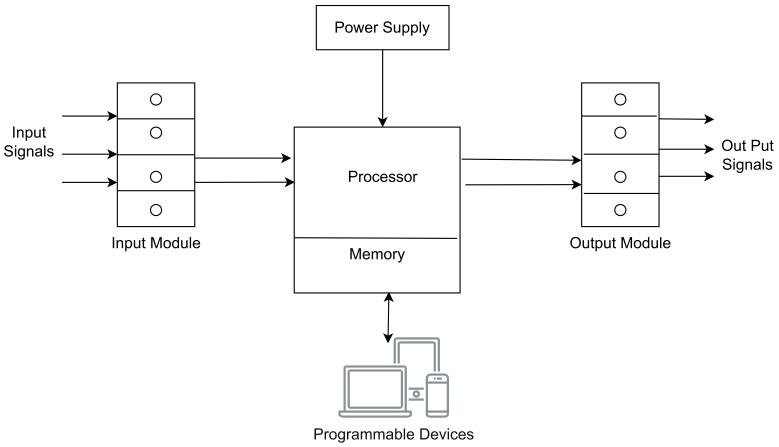
Components of a PLC system.

Figure 8 below shows the components of a PLC system.

- Power Supply Unit: The power requirements are contingent upon the particular type of PLC employed in the application. This unit converts AC to DC voltage suitable for PLC. This unit comprises short-circuit protection switches at all levels, control transformers, switching power supply, and other components [38];
- Processor or CPU: This component includes a microprocessor, system memory, serial communication ports, and a LAN connection. A power supply may also be included in specific cases to deliver the necessary power to the CPU;
- Input/Out modules: Input and output modules serve as the connection points between the control environment’s field devices (comprising both input and output equipment) and the processor. The input devices encompass sensors, push buttons, limit switches, and similar items, while the output devices consist of motors, relays, solenoid valves, and the like. I/O devices can be broadly categorized into two groups: discrete or digital modules and analog modules;
- Programmable devices: As seen in Figure 8 above, Programming tools are utilized to load the specific program into the CPU’s memory. We can develop our program using a widely recognized and user-friendly language called ladder logic.

## 3. ICS System Design Considerations

While Section 2 introduced the essential components and functions of Industrial Control System technologies, designing an ICS, including whether a SCADA, DCS, or PLC-based topology is used, depends on many factors. This section identifies key factors that drive design decisions regarding the ICS’s control, communication, reliability, and redundancy properties [39]. These factors also help to determine the security needs of the system.

- Control Timing Requirements: Within ICS, a spectrum of time-related demands exists, encompassing the need for rapid responses, consistency, regularity, and synchronization. These requirements can present difficulties for humans in consistently and reliably meeting them, emphasizing the importance of implementing automated controllers. In certain specific situations, it becomes vital for computations to occur close to sensors and actuators to minimize communication delays and ensure the timely execution of essential control functions;
- Safety: The inclusion of safety requirements in the system’s design is of utmost importance. Systems must possess the ability to detect unsafe conditions and take measures to transform them into safe conditions. In numerous safety-critical operations, the presence of human oversight and control within potentially hazardous processes remains an indispensable element of the safety system;
- Geographic Distribution: The level of distribution in systems can exhibit a broad spectrum, ranging from compact systems such as local PLC-controlled processes to far-reaching, extensively distributed systems such as oil pipelines and the electric power grid. A higher degree of distribution often necessitates using wide-area communication methods such as leased lines, circuit switching, packet switching, and mobile communication solutions;
- Impact of Failures: Failures in control functions can lead to varying consequences in different domains. Systems with more significant impacts often necessitate the ability to sustain operations through redundant controls or the capacity to operate in a reduced-capability state. The design must specifically cater to these requirements;
- Hierarchy: To create a central hub that can gather data from diverse locations, supervisory control is utilized, enabling control decisions to be made based on the system’s current status. Hierarchical or centralized control is commonly employed to provide human operators with a holistic view of the entire system;
- Control Complexity: Simple controllers and predetermined algorithms are often sufficient for managing control functions. Nevertheless, in highly complex systems such as air traffic control, the presence of human operators becomes essential to ensure that all control actions align with the overarching objectives of the system;
- Availability: The system’s dependability, indicating its accessibility, remains critical during the design phase. Systems requiring exceptional availability or continuous operation might require heightened redundancy or alternative communication and control components strategies.

## 4. ICS Cyber Attack Incidents

In year 2020, the Cybersecurity and Infrastructure Security Agency detailed four primary priorities that served as the central focus of its endeavors to reduce cyber risks within control systems [40].

- Protecting ICS environments from the most critical threats is an absolute necessity;
- It is vital to preserve operational resilience by addressing systemic weaknesses and fortifying the capacity of control systems to withstand cyber incidents with minimal adverse effects on critical infrastructure;
- We must provide critical infrastructure owners, operators, and cybersecurity defenders with the necessary technologies and tools to significantly increase the time, costs, and technical hurdles for adversaries;
- Identify and proactively counteract adversaries, preempting any potential harm. CISA and its partners will cooperate to improve visibility within OT environments, guaranteeing swift detection and elimination of malicious activity before it can cause widespread damage.

### 4.1. Recent Attacks on ICS

In 2021, the actual threats confronting ICS garnered increased attention. The number of vulnerabilities identified in operational technology (OT) devices and their corresponding management systems witnessed a growth of over 50%. Concurrently, ransomware groups continued to target manufacturing and critical infrastructure with persistence. The reported vulnerabilities in 2021 surged by 52%, reaching almost 1440, in contrast to the prior year [41]. Cyber attacks targeting industrial facilities result in widespread repercussions [42]. Frequently, threat actors focus on Industrial Control Systems (ICS) to execute these attacks, leading to complete or partial shutdowns of crucial facilities, financial losses, data breaches, and potential health hazards [43]. In 2022, there was a significant surge in ICS, with an 87% increase in ransomware attacks targeting industrial organizations and a 35% rise in the number of ransomware groups focusing on industrial control and operational technology (OT) systems [44]. Based on the cyber incidents witnessed globally, it has become evident that threat actors’ technical capabilities have evolved substantially. Equally concerning is their readiness to cause physical harm [45]. Below are some of the recent most significant cyber attacks on industrial facilities that disrupted government and non-government facilities.

#### 4.1.1. Colonial Pipeline—Ransomware Attack

The cyberattack that occurred on 7 May 2021, targeting Colonial Pipeline, gained worldwide attention due to its far-reaching consequences, including a severe fuel shortage and skyrocketing prices. The breach transpired when hackers managed to access the company’s network by exploiting an inactive virtual private network (VPN) account that had remote access to their computer system. To regain control of their network, Colonial Pipeline ultimately had to pay USD 4.4 million to the DarkSide hacker group in exchange for the decryption tool required for network restoration [46].

#### 4.1.2. CPC Corp. Taiwan—Ransomware

In May 2020, CPC Corp, Taiwan’s state-owned petroleum and natural gas company, encountered a ransomware incident that left its payment system unusable. The attackers used a USB flash drive to breach the company’s computer network. While it did not affect oil production, it did disrupt CPC Corp’s payment card system. This cyberattack was attributed to the Winnti Umbrella group, a China-linked entity known for its targeting of software companies and political organizations [47].

#### 4.1.3. Triton (2017)—Malware

During the incident in 2017, a Russian agency utilized Triton to specifically focus on a Schneider Electric Triconex safety instrumented system (SIS), which holds the responsibility of initiating safe shutdown protocols during emergencies. Following their initial access, the attackers then traversed through both IT and OT networks until they reached the safety system, where they introduced the Triton malware. This malicious software made alterations to the in-memory firmware, introducing harmful code. The FBI cautioned that this situation could have led to damage to the facility, system disruptions, or even potential loss of life if the SIS had failed to carry out the safe shutdown procedures [48,49].

#### 4.1.4. Ukraine Power Grid Hack—Malware Attack

On 23 December 2015, a power outage that impacted approximately 1.4 million residents of Ukraine was linked to the espionage Trojan called Black Energy. This incident appears to represent the first instance of malware being employed to facilitate a widespread power disruption.

#### 4.1.5. SFMTA Ransomware Attack

On 25 November 2016, a malware infection struck approximately 2000 of the San Francisco Municipal Transport Authority’s (SFMTA) 8000 computer systems. This malware also managed to compromise physical ticketing machines, leading to the SFMTA offering free rides to passengers over the Thanksgiving weekend. Additionally, Muni bus drivers were forced to create handwritten route assignments. The agency expected to incur a daily revenue loss of around USD 559,000 during the period when they were unable to collect fares [50].

Table 1 below provides an overview of potential ICS threat events and their corresponding descriptions.

## 5. Vulnerability of Industrial Control System(ICS)

Remote attacks often serve as the initial point of entry for targeting ecosystems of devices. Attackers take advantage of known vulnerabilities in specific protocol implementations, using readily available pre-written attack scripts, simplifying the attack process [51]. By monitoring non-encrypted traffic, attackers can gather valuable information about the system, enabling them to escalate the attack and eventually gain control over the targeted device. This emphasizes the significance of putting in place robust security measures, including encryption and routine vulnerability assessments, to fortify defenses against cyber threats and ensure the protection of critical industrial systems [52]. Indeed, attackers targeting industrial control systems rely on exploiting one or more existing vulnerabilities [53]. These vulnerabilities can stem from various areas, including:

- Architecture and Design Vulnerabilities: Deficiencies in the overarching system architecture and design can be leveraged by malicious actors to obtain unauthorized access or manipulate control processes;
- Configuration and Maintenance Vulnerabilities: Incorrect or inadequate system configurations and poor maintenance practices can create opportunities for attackers to compromise the system’s security;
- Physical Vulnerabilities: Physical access to industrial control systems can lead to potential exploits, such as unauthorized tampering with hardware or gaining direct access to critical components;
- Software Development Vulnerabilities: Errors or flaws in software development can introduce vulnerabilities that attackers may exploit to infiltrate and compromise the system;
- Communication and Network Configuration Vulnerabilities: Insecure communication protocols and improperly configured networks can provide attackers with entry points to intercept or manipulate data and control commands.

### 5.1. Architecture and Design Vulnerabilities

(a) Inadequate incorporation of security into architecture and design: Incorporating security into the architecture and design of ICS should be considered from the outset, considering the budget and schedule constraints of the ICS project [54]. The security architecture should be integral to the overall Enterprise Architecture [55]. To effectively address security concerns, the ICS architecture must encompass several essential aspects, including:
  - Identification and Authorization of Users: Robust authentication mechanisms should be implemented to verify the identity of users accessing the ICS. Authorization procedures must guarantee that users are allocated appropriate privileges in accordance with their designated roles and responsibilities;
  - Access Control Mechanism: Access control policies and mechanisms should be implemented to restrict and manage users’ access to critical components and functions within the ICS. This ensures that only authorized personnel can interact with specific system elements;
  - Network Topology: The network architecture of the ICS should be designed with security in mind. Network segmentation, firewalls, and other security measures should be deployed to prevent unauthorized access and isolate critical components from less secure areas;
  - System Configuration and Integrity Mechanisms: Implementing system configuration controls and integrity mechanisms ensures that the ICS operates within specified parameters and that any unauthorized changes or tampering are promptly detected and addressed.
  By proactively integrating these security considerations into the ICS architecture and design, organizations can build resilient and secure systems that protect against potential cyber threats and ensure the continuity and safety of critical industrial processes.
(b) The insecure architectural design permitted to evolve: The network infrastructure of ICS has frequently evolved and adapted to meet business and operational needs, often without sufficient consideration of the potential security consequences of these alterations. As a result, security vulnerabilities may have unintentionally emerged in certain parts of the infrastructure. In the absence of corrective measures, these vulnerabilities could serve as potential points of unauthorized access into the ICS.
(c) No security perimeter defined: Without a clearly defined security perimeter for the ICS, it becomes challenging to guarantee the proper deployment and configuration of essential security measures [56]. This situation can result in unauthorized access to systems and data, along with other potential issues.
(d) Inadequate collection of event data history: Investigative analysis relies on the gathering and preservation of an ample amount of data. In the absence of thorough and precise data collection, determining the cause of a security incident may become exceedingly difficult or even impossible [57]. Security incidents have the potential to go undetected, leading to additional harm and disruption. Consistent security monitoring is equally crucial for identifying issues related to security controls, such as misconfigurations and malfunctions.

### 5.2. Configuration and Maintenance Vulnerabilities

(a) Operating system (OS) and application security patches are not maintained or vendor declines to patch vulnerability: Outdated operating systems and applications may house newly uncovered vulnerabilities that could be taken advantage of. It is of utmost importance to create documented guidelines for overseeing security patch management. In situations where unsupported ICS operating systems are utilized, access to security patch support may not be available. Consequently, these procedures should also incorporate backup plans for addressing vulnerabilities that may never receive prompt patch updates.
(b) OS and vendor software patches may not be developed until a considerable time after security vulnerabilities are initially discovered: Due to the close integration between ICS software and the underlying ICS infrastructure, any modifications must undergo extensive and time-consuming regression testing, incurring significant costs. The duration required for this testing and the subsequent distribution of updated software can create a prolonged window of vulnerability.
(c) The installation of malicious software, known as malware, is a prevalent form of attack: Installation of malicious software, or malware, is a common attack. Malware protection software, such as antivirus software, must be kept current in a dynamic environment. Outdated malware protection software and definitions open the system to new malware threats.
(d) Insufficient examination of security modifications: Introducing modifications to hardware, firmware, and software without conducting prior testing poses a risk to the smooth operation of the ICS. It is imperative to establish well-documented procedures for evaluating the security ramifications of any changes. It is vital to refrain from using operational systems in live environments for testing. Additionally, the testing of system modifications may necessitate collaboration and coordination with system vendors and integrators.
(e) Inadequate remote access management: There are various reasons why remote access may be required for an ICS, such as system maintenance tasks performed by vendors and system integrator or ICS engineers accessing geographically distant system components. To safeguard against unauthorized access, it is essential to maintain robust control over remote access capabilities.
(f) Critical configurations are neither stored nor subjected to backup procedures: Procedures for restoring ICS configuration settings should be easily accessible to address unintentional or malicious configuration alterations, ensuring system availability and data protection. It is imperative to establish thorough and meticulously documented procedures for maintaining ICS configuration settings.
(g) Unsecured information stored on portable devices: The security of the system could be compromised if confidential information, such as passwords and dial-up numbers, is stored without encryption on portable devices such as laptops and mobile devices. To minimize this risk, it is essential to implement policies, procedures, and mechanisms to safeguard this data.
(h) Passwords generated, utilized, and safeguarded in a manner inconsistent with established policies: The extensive knowledge about password management in IT is relevant to ICS. Maintaining effectiveness requires strict compliance with password policies and procedures. Departures from these guidelines can considerably increase vulnerabilities in ICS.
(i) Insufficient access controls implemented: Access controls should align with how the organization assigns responsibilities and privileges to its personnel. Poorly defined access controls can grant an ICS user too many privileges or restrict them excessively.
(j) The absence of configuration management for hardware, firmware, and software can give rise to significant challenges: The organization may lack visibility into its inventory, the versions in use, their locations, or their patch status, resulting in an inconsistent and ineffective security posture. To safeguard an ICS against inadequate or improper modifications at all stages, including before, during, and after system implementation, it is essential to establish a structured process for controlling changes to hardware, firmware, software, and documentation. Failing to institute configuration change management procedures can introduce security oversights, vulnerabilities, and risks. To comprehensively secure an ICS, maintaining an accurate inventory of system assets and their current configurations is imperative [58]. These processes are essential for the implementation of business continuity and disaster recovery plans.
(k) Improper data linking: Data storage systems within ICS can be connected to non-ICS data sources. One such example is database links, which facilitate the automatic replication of data from one database to others. However, incorrect configuration of data linkage can introduce vulnerabilities, potentially enabling unauthorized access to or manipulation of data.
(l) Malware protection deployed without thorough testing: If malware protection software is deployed without adequate testing, it has the potential to disrupt the normal operation of the ICS and hinder the system’s ability to carry out essential control actions [59].
(m) Denial of service (DoS): ICS software might be prone to DoS attacks, leading to the obstruction of authorized access to a system resource or the disruption of system operations and functions [60,61].
(n) Lack of installed intrusion detection/prevention software: Incidents can result in system availability and integrity loss, data capture, modification, and deletion, and incorrect execution of control commands. IDS/IPS software may stop or prevent various types of attacks, including DoS attacks, and also identify attacked internal hosts, such as those infected with worms. IDS/IPS software must be tested before deployment to determine that it does not compromise the normal operation of the ICS.
(o) Lack of log maintenance: In the absence of accurate logs, pinpointing the cause of a security incident can become a challenging task.
(p) Unauthorized personnel have physical access to equipment: Limited physical access to ICS equipment should be exclusively granted to essential personnel, while considering safety prerequisites such as emergency shutdowns or restarts. Inappropriate access to ICS equipment can result in any of the following consequences:
  - Theft of data and hardware;
  - Physical harm or destruction of data and hardware;
  - Unauthorized alterations to the operational environment (e.g., data connections, unauthorized utilization of removable media, addition/removal of resources);
  - Disconnection of physical data links;
  - Untraceable interception of data (including keystroke and other input logging).
(q) Radio frequency, electromagnetic pulse (EMP), static discharge, brownouts, and voltage spikes: Control systems hardware is susceptible to various threats, including radio frequency interference,EMP, static discharges, brownouts, and voltage spikes [62]. The consequences can vary from temporary disruption of command and control to irreversible harm to circuit boards. It is advisable to implement adequate shielding, grounding, power conditioning, and surge suppression measures.
(r) Lack of backup power: In the absence of backup power for essential assets, a widespread power outage can result in the ICS shutdown, potentially creating a hazardous situation. Additionally, the loss of power could result in the activation of insecure default settings.
(s) Physical ports lacking security measures: Unprotected USB and PS/2 ports could permit unauthorized connections, including thumb drives and keystroke loggers [63].

### 5.3. Software Development Vulnerabilities

(a) Inadequate data validation: ICS software might fail to effectively validate user inputs or incoming data for accuracy, potentially leading to various vulnerabilities. These vulnerabilities encompass issues like buffer overflows, command injections, cross-site scripting, and path traversals.
(b) Installed security features remain inactive in their default settings: The security features bundled with the product become ineffectual unless they are actively activated or, at a minimum, acknowledged as disabled.

### 5.4. Communication and Network Configuration Vulnerabilities

(a) Unused data flows: Data flow controls are essential based on the attributes of the data, as they help regulate the permissible transfer of information between systems. These controls play a crucial role in preventing data exfiltration and unauthorized operations.
(b) Inadequate firewall and router logs: In the absence of precise and comprehensive logs, identifying the root cause of a security incident might become an insurmountable challenge.
(c) Standard, well-documented communication protocols are used in plain text: Adversaries with the ability to monitor ICS network activity can exploit the lack of encryption in certain protocols. Protocol analyzers and other utilities can be utilized to decode data transferred over protocols such as Telnet, File Transfer Protocol (FTP), Hypertext Transfer Protocol (HTTP), and Network File System (NFS). Since these protocols do not employ encryption, the data transmitted is easily readable by anyone monitoring the network. This exposes sensitive information, including login credentials and commands sent between devices, potentially leading to unauthorized access and manipulation of the ICS network. Adversaries can leverage this vulnerability to perform attacks against the ICS, such as eavesdropping, session hijacking, and man-in-the-middle attacks. By exploiting the lack of encryption, they can manipulate ICS network activity, disrupt operations, and potentially cause significant harm to industrial processes and critical infrastructure. To counter these threats, it is crucial to implement secure communication protocols, such as Secure Shell (SSH) and Secure Socket Layer/Transport Layer Security (SSL/TLS), that encrypt data transmissions and protect against unauthorized access and manipulation of ICS network activity. Employing robust authentication mechanisms and regular security assessments also enhances the overall security posture of the ICS environment.
(d) Firewalls are either absent or configured incorrectly: Insufficiently configured firewalls can lead to unrestricted data flow between diverse networks, such as control and corporate networks. This situation can create openings for potential attacks and the spread of malware across networks, ultimately exposing sensitive data to potential monitoring, eavesdropping, and allowing unauthorized access to systems [64].
(e) Authentication of users, data, or devices is either inadequate or entirely absent: Numerous ICS protocols lack authentication at any level. In the absence of authentication, there exists the possibility of data or device manipulation, replay attacks, and spoofing of elements like sensors and user identities.
(f) Absence of communication integrity verification: Most industrial control protocols lack built-in integrity checks, potentially allowing adversaries to tamper with communications without detection. To ensure integrity, ICS systems can implement lower-layer protocols like IPsec, which provide data integrity protection.
(g) Insufficient authentication measures between wireless clients and access points: It is essential to establish robust mutual authentication between wireless clients and access points to prevent clients from connecting to rogue access points deployed by adversaries. Additionally, this authentication ensures that adversaries cannot connect to any of the ICS’s wireless networks.

## 6. ICS Communication Protocols Cyber Vulnerabilities

**Figure 9:**
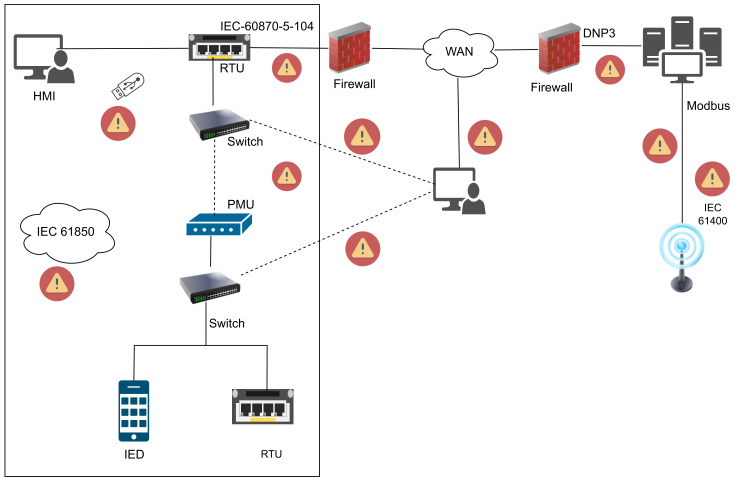
Vulnerabilities of industrial protocols under an application scenario.

With the existing system integration, the primary function of ICS is to gather real-time data, realize device automation, and supervise the entire system [65]. This is achieved through a number of communication protocols, including but not limited to DNP3, Modbus, IEC 60870-5-104, IEC 61400-25,IEEE C37.118, Message Queuing Telemetry Transport (MQTT), and Open Platform Communications(OPC). In this section, we analyze vulnerabilities of industrial protocols under an application scenario, as shown in Figure 9.

**Figure 10:**
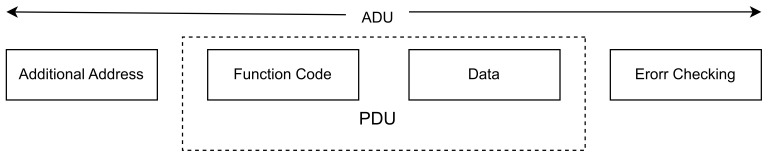
General Modbus framework.

(a) Distributed Network Protocol 3 (DNP3): DNP3 is an application layer protocol with a multi-tier structure, primarily utilized in smart grid applications [66]. DNP3 is an internationally recognized standard created to ensure dependable data transmission and support functionalities for ICS. DNP3 incorporates the Enhanced Performance Architecture (EPA), a streamlined version derived from the OSI reference model, offering significant workload reduction. DNP3 typically operates over the Transmission Control Protocol (TCP) and is assigned to port 20000. It adheres to a client–server model, involving two distinct entities: the master, which performs client functions, and the slave or outstation, which carries out server functions. The primary purpose of the slave or outstation is to respond to requests initiated by the master [67]. In this model, the master is empowered to supervise, regulate, and collect data from slaves, thereby facilitating comprehensive control over the production processes [68]. It is divided into three layers, namely:
  - The Data Link layer: It is responsible for sending and receiving frames and contains header information such as source DNP3 address and destination DNP3 address. At the same time, it is also responsible for calculating errors through Cyclic Redundancy Check (CRC) and checking the link’s status;
  - The Transport layer: The main purpose for this layer lies in the fragmentation of large packets received by the Application layer, while its header contains the information required to reassemble the fragments;
  - The Application layer: This layer creates the message to be communicated; however, this layer’s header differs depending on whether the message creator is a master or a slave, as the latter’s header contains the Internal Indications field to better describe the node’s status.
(b) Modbus: The Modbus protocol, initially created in 1979 by the American company Gould-Modicon, is an openly available communication standard designed for enabling the communication of programmable logic controllers. With Modbus, a master device can efficiently exchange data with multiple slave devices. While theoretically, every node on the network can transmit messages, it is most common for communication to be instigated by the master device [69]. The Modbus protocol has become the most widespread protocol for communication between control devices and industrial automation. The Modbus was developed especially for industrial applications, public domain, and with no royalties charged, easy to use and maintain, while enabling Bit and word communication between devices of different manufacturers without restrictions [70]. The protocol adopts a unique protocol data unit (PDU) different from ordinary architectures. The Modbus mapping on a particular bus can bring in extra fields to the application data unit (ADU). Figure 10 below illustrates the concrete format of the general message frame of a Modbus.

**Figure 11:**
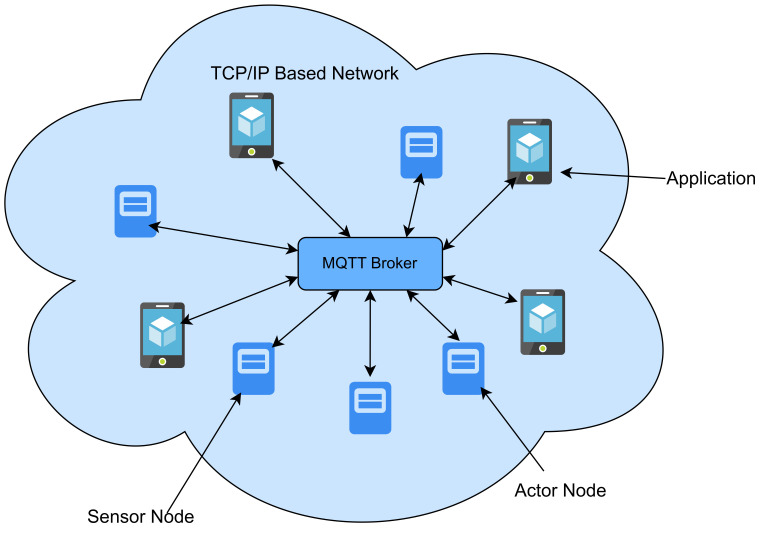
MQTT protocol design.

**Figure 12:**
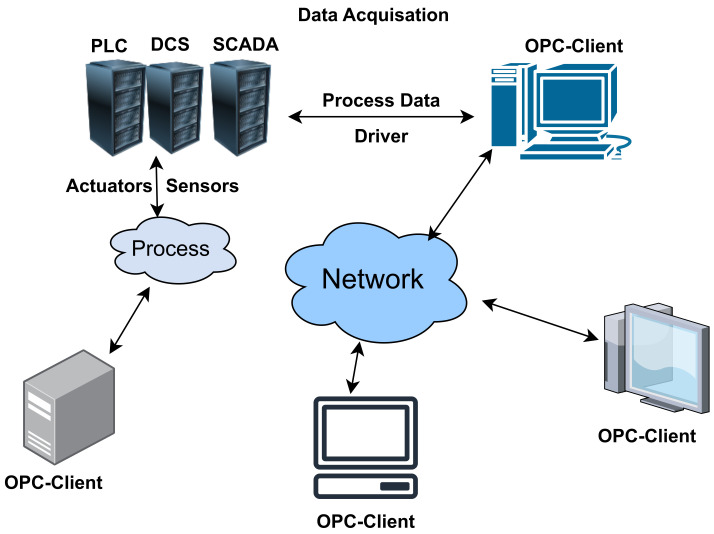
OPC protocol design.

They are three primary variations of the Modbus protocol, as seen below:

(a) Modbus RTU: Modbus RTU is a widely used communication protocol in industrial automation and control systems. It is part of the Modbus family of protocols and is designed for serial communication over RS-232 or RS-485 interfaces. Modbus RTU is known for its simplicity and efficiency in transmitting data between devices such as PLCs, HMIs, sensors, and other industrial equipment [71].
  Key features and characteristics of Modbus RTU include:
  - Modbus RTU uses serial communication, which is well-suited for industrial environments. It can be transmitted over RS-232 or RS-485, allowing for long-distance communication and noise immunity;
  - Modbus RTU follows a master–slave architecture, where a master device (e.g., a PLC or HMI) initiates requests, and slave devices (e.g., sensors or actuators) respond to those requests. This architecture enables centralized control and data acquisition;
  - Communication in Modbus RTU is based on frames or packets. Each frame includes a start bit, address, function code, data, and error-checking (CRC or LRC). The structure is designed for simplicity and ease of implementation;
  - Modbus RTU supports various data types, including binary (coils), discrete inputs, input registers, and holding registers, allowing for the exchange of different types of data;
  - The master device typically polls slave devices by sending requests for data. This polling mechanism allows the master to request specific information from each slave device;
  - Modbus RTU is known for its efficiency and speed in data transmission. It is suitable for real-time control and monitoring applications in industrial settings.
(b) Modbus ASCII: Modbus ASCII is another variant of the Modbus communication protocol used in industrial automation and control systems. Like Modbus RTU, it is designed for serial communication, but it employs a different encoding format [72].
  Key characteristics of Modbus ASCII include:
  - Modbus ASCII represents data using ASCII characters, making it more human-readable than Modbus RTU. Each 8-bit byte of data is converted to 2 ASCII characters;
  - Modbus ASCII is character-oriented, and each character is transmitted as a single byte (8 bits). This makes it more suitable for systems where ASCII-based communication is preferred;
  - Modbus ASCII devices can often communicate with Modbus RTU devices with proper configuration and protocol translation. This compatibility allows for flexibility when integrating different devices;
  - The ASCII format of Modbus ASCII frames makes it human-readable, which can be advantageous for troubleshooting and debugging purposes;
  - Modbus ASCII has a higher overhead compared to Modbus RTU due to the character-based encoding. This can result in slower data transfer rates, which may not be suitable for real-time applications.
(c) Modbus TCP: Modbus TCP uses the more modern Ethernet communication protocol and is frequently employed in industrial automation and control systems to establish network connections with devices such as PLCs, HMIs, and sensors [73]. Below are some key features and characteristics of Modbus TCP
  - Modbus TCP operates over standard Ethernet networks, allowing for fast and efficient data transmission. It is well-suited for modern industrial environments and can be used alongside traditional office IT networks;
  - Modbus TCP follows a client–server architecture. In this setup, client devices (typically master devices such as PLCs or HMI) request data from server devices (slave devices), and the server responds with the requested information. This architecture allows for distributed control and monitoring;
  - Modbus TCP uses standard TCP/IP communication protocols. It relies on the widely used Transmission Control Protocol (TCP) to establish connections and ensure reliable data transfer;
  - Unlike Modbus RTU and Modbus ASCII, which use character-based frames, Modbus TCP uses binary frames. Each frame consists of a transaction identifier, protocol identifier, length field, unit identifier, function code, and data. This binary format allows for efficient data transmission;
  - Modbus TCP offers high-speed data transmission, making it suitable for real-time control and monitoring applications. Ethernet’s speed and efficiency contribute to the quick exchange of data;
  - Modbus TCP is widely used in various industries and is considered a standard for Ethernet-based communication in industrial automation and control systems;
  - Similar to other Modbus variants, Modbus TCP is an open and standardized protocol, allowing devices from various manufacturers to communicate as long as they adhere to the protocol specifications;
  - Devices using Modbus TCP communicate based on IP addresses, making it possible to have devices distributed across a network or even connected over the internet.
  A group of researchers proposed the Modbus/TCP Security protocol, which incorporates authentication and authorization mechanisms to ensure protection against deliberate unauthorized access as an enhancement for improved security in the ICS framework [74].
(d) IEC 60870-5-104: It is an unencrypted protocol, meaning it transmits data in plain text without any authentication mechanism over TCP/IP.
  IEC-60870-5-104 is an international standard providing communication standards between the SCADA system and substations. In transmission, the application layer of this protocol conveys an application service data unit(ASDU).
(e) IEC 61850: It is a collection of communication norms that outline protocols for designating devices, data, and communication systems linked to the automation of electric power substations [75].
  The IEC 61850 standard presents guidelines for establishing best practices in substation engineering, encompassing protection, monitoring, integration, metering, testing, and control. Within the domain of substation automation, the need for high-speed communication is imperative to meet the data transfer rates required by modern automatic control and monitoring systems (source: [76]).
  The IEC 61850 standard delivers services, protocols, and a structure engineered to streamline the modeling and communication of Intelligent Electronic Devices (IEDs) and supervisory equipment in power system automation [77].
  The substation communication system is divided into three layers by IEC 61850: the process bus, the interval, and the station.
  - Process Layer: The process layer includes various primary equipment and intelligent electronics components, realizing the major functions of smart substations. The process layer within the substation involves gathering data from transformers and transducers that are interconnected with the primary power system process [78];
  - Interval Layer: This includes secondary devices like relay protection equipment and control devices and functions as a barrier between the other two layers, guaranteeing safe and dependable operations;
  - Station Control Layer: This layer primarily handles the monitoring and management of the intelligent station, enabling comprehensive measurement and control capabilities for the entire station.
(f) IEC 61400-25: This protocol is specially designed to communicate the wind farm supervisory system and is an extension of the IEC 61850 standard in wind power generation. The basic purpose of this protocol is to provide network communication standards between the wind farm supervisory system and other subsystems and to realize the equipment’s interoperability with different manufacturers. IEC 61400-25 interface uses MMS and web service for remote supervisory control at wind power plants [79].
(g) IEEE C37.118: In substations, this protocol is commonly used to establish synchronization and define the standards for data transmission formats. It outlines four distinct message types: data, header, configuration, and command. In the typical transmission process, these messages are converted into frames, and the PMU exclusively transmits data frames to other devices.
(h) Message Queuing Telemetry Transport(MQTT): MQTT is a lightweight publish-subscribe messaging protocol designed for low-bandwidth, high-latency, or unreliable networks [80]. It is widely used in IoT applications for real-time data communication between devices and systems [81]. MQTT’s lightweight nature makes it suitable for resource-constrained devices. MQTT can be vulnerable to eavesdropping, man-in-the-middle attacks, and unauthorized access if not properly secured. Robust security mechanisms, and security considerations are often implemented at the application level, which include using TLS/SSL for encryption and username/password authentication. The Figure 11 below illustrates the MQTT protocol design.
(i) Open Platform Communications (OPC): OPC is a set of standards for industrial communication, and it plays a crucial role in Industry 4.0, which focuses on the automation and digitization of manufacturing processes [82]. OPC enables the interoperability of devices, equipment, and systems in industrial environments. It includes various specifications, such as OPC Data Access (DA) and OPC Unified Architecture (UA). Some of the common threats include data interception, unauthorized access, and denial-of-service attacks. OPC UA, in particular, has robust security features, including authentication, encryption, and authorization, making it suitable for secure industrial communication [83]. Figure 12 below illustrates the general OPC protocol design.

### Cybersecurity Issues Related to the Discussed Protocols

Given that the ICS integrates both cyber and physical subsystems, it possesses inherent vulnerabilities that render the system susceptible to attacks from both internal and external sources [84]. Every form of threat can have catastrophic consequences for power systems. It’s widely acknowledged that cybersecurity typically involves aspects such as authentication, authorization, encryption, confidentiality, integrity, and availability.

- Confidentiality: Unauthorized individuals can exploit this vulnerability to access confidential information about the ICS for illicit purposes;
- Availability: In the event of availability loss, the system could forfeit its capacity to maintain control, resulting in substantial economic losses;
- Integrity: When a missing or corrupted data packet is received, it renders the entire transmission process ineffective, causing significant disruptions to normal operations;
- Authorization and Authentication: Malicious actors may exploit this vulnerability by manipulating the function code to send arbitrary data to others, ostensibly under the guise of seeking constructive feedback. Prominent protocols lack authentication mechanisms for verifying identity, making it easy for unauthorized parties to obtain privileges and forge protocol packets.

The known vulnerabilities in protocols are examined, and the corresponding weaknesses are identified and documented accordingly; see Table 2 below.

## 7. Existing Measures to Strengthen the Cybersecurity of Industrial Control Systems

**Figure 13:**
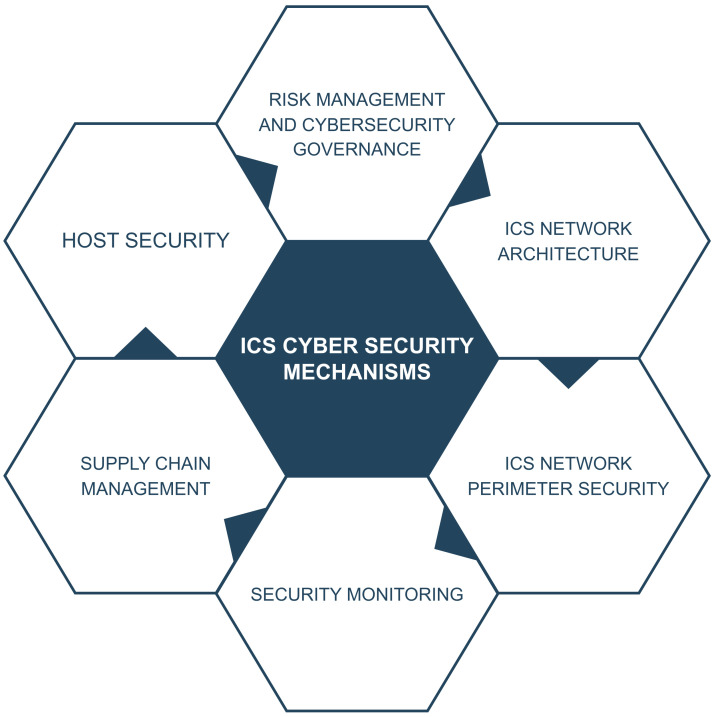
Countermeasure for defending ICSs.

The Industrial Control system should not only be able to deal with known attacks but also be resilient against any possible evasion tactics [39]. With the increasing number of incidents reported to the ICS-Cyber Emergency Response Team (CERT), including those that go unreported or undetected, there is increasing frequency and complexity in our adversaries. Securing ICSs against the modern threat requires well-planned and well-implemented strategies that will provide network defense teams with a very effective way to detect, counter, and expel an adversary, preserving the critical process and business continuity of industrial control systems [91]. In this section, we study the strategic countermeasures against cyber attacks for industrial control systems in detail, as shown in Figure 13 below.

### 7.1. Risk Management and Cybersecurity Governance

- Identify threats to the organization, which generally includes the steps of inventorying system elements, defining metrics (how to measure the level of risk), and the threats are taken into account;
- Maintain ICS asset inventory of all hardware, software, and supporting infrastructure technologies, which makes it possible to establish a list of the company’s critical assets and processes. The impact analysis is carried out by examining the consequences on each of the security objectives: availability, integrity, and confidentiality [92];
- Develop cybersecurity policies, procedures, training, and educational materials that apply to the organization’s ICS available on Cybersecurity and Infrastructure Security Agency [93];
- Organizations should embrace adaptive cybersecurity measures for critical infrastructure by evolving policies beyond mere tools for enforcing predefined security requirements. These policies should become adaptive entities capable of responding and evolving in the face of emerging threats and attacks [94];
- Develop and practice incident response procedures that join IT and OT response processes.

### 7.2. ICS Network Architecture

**Figure 14:**
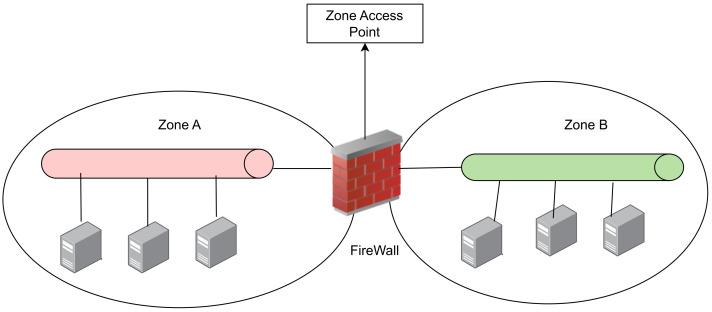
Network segmentation or zoning.

- Implement network segmentation whenever feasible, categorizing systems into network zones according to their roles, significance to the business, risk profiles, or other criteria established by the organization. To accomplish this, employ a filtering device like a packet filtering or stateful inspection firewall at the entry point of each zone. Ensure that each zone adheres to a clearly defined baseline, consistently applied to all systems within that specific zone [95]. A network zone should always have one entry point, as depicted in Figure 14 below;
- Design a network topology for ICS that incorporates multiple layers, prioritizing the most crucial communications within the most secure and dependable layer [96];
- The cost of a total system failure can be catastrophic in ICS. The use of true data diodes utilize proprietary software to control data flow and allow one-way network traffic to be handled properly [97]. Data diodes and unidirectional gateways are engineered to block reverse communications at the physical layer, often employing a single fiber-optic connection represented by a single fiber strand. The ”transmit” component typically does not incorporate “receive” circuitry, while the “receive” component lacks “transmit” capabilities. This configuration guarantees absolute physical layer security but sacrifices bidirectional communication [98];
- Establish demilitarized zones (DMZs) to configure a physical and logical subnetwork that serves as an intermediary for connected security devices, preventing direct exposure [99].

### 7.3. ICS Network Perimeter Security

- Configure firewalls to control traffic between the ICS and corporate IT networks. Firewalls are network devices created to monitor and inspect incoming and outgoing traffic. They provide a layer of defense between networks. A set of rules, or access control lists (ACLs), can be established to allow or block certain packets between those networks [100];
- Utilize IP geo-blocking that enables blocking outgoing and incoming network connections based on a geographic location [101]. This technology relies on devices’ IP addresses and other identifying factors. IP filtering is sometimes used as a security tool to protect from certain types of hackers [102,103];
- Use jump servers as a central authorization location between ICS network security zones. These measures aid in achieving network isolation between segments with varying security levels. Jump servers are sometimes used in conjunction with additional security tools such as firewalls and Intrusion Detection Systems (IDS) to create an exceptionally secure environment in alignment with the Defense-In-Depth concept [104];
- Prohibit remote and ongoing vendor or employee access to the control network, including the use of backdoor passwords and maintenance accounts. Manufacturers should disclose in written documentation if they employ any such accounts [105];
- Catalog and monitor all remote connections to the network. While playing important roles in the ICS context, PLCs and RTUs lack adequate security mechanisms to overcome buffer overflow exploits or man-in-the-middle and a wider array of other cyber attacks [106,107]. The author’s suggested Shadow Security Unit (SSU) is connected in parallel to RTU/PLCs, allowing it to capture and decode the SCADA protocol data flow. It then correlates this data with the status of the physical I/O modules that communicate with sensors and actuators in the field. This makes it feasible to establish a redundant security-checking mechanism that adopts a “black box” approach when assessing the behavior of the monitored devices [108].

### 7.4. Security Monitoring

- Measuring the baseline of normal operations and network traffic for ICS researchers have proposed a method using machine learning combined with passive monitoring and a priori knowledge of protocols used. It is important that no measuring device or monitoring system interferes with the ICS environment under scrutiny [109];
- Configure Intrusion Detection Systems (IDS) to create alarms for any ICS network traffic outside normal operations;
- Track and monitor audit trails on critical areas of ICS. Set up a Security Information and Event Management system (SIEM) to gather pertinent data from various origins, detect variances from established norms, and execute suitable responses [110];
- Establish a SIEM system to oversee, analyze, and correlate event logs throughout the ICS network for the detection of intrusion attempts [111].

### 7.5. Host Security

- Promote a culture of patching and vulnerability management. Patch management reduces cybersecurity risks and ensures production availability [112]. Smart prioritization is a method for sequencing patches in a complex, interconnected network, consisting of three fundamental steps. It seamlessly integrates principles from system modeling, risk assessment, and game theory. Smart prioritization makes use of existing knowledge, insights, and previous experiences related to system dynamics to identify an efficient and exceptionally effective defensive strategy [113];
- Test all patches in off-line test environments before implementation;
- Implement application whitelisting on human–machine interfaces. Application whitelisting is a security technique that enhances security by allowing systems to run only those applications that have been explicitly approved and listed in a designated whitelist [114];
- Harden field devices, including tablets and smartphones;
- Replace out-of-date software and hardware devices;
- After conducting thorough testing to confirm that it will not disrupt ICS operations, disable unused ports and services on ICS devices;
- Implement and test system backups and recovery processes;
- Configure encryption and security for ICS protocols.

## 8. Machine Learning Integration in Defending ICS from Cyber Attacks

Machine learning, a branch of artificial intelligence (AI) and computer science, centers on utilizing data and algorithms to mimic the learning process of humans, with the aim of progressively enhancing its accuracy [115]. Applying machine learning in cybersecurity makes the malware detection process more actionable, scalable, and effective than traditional approaches, which require human intervention [116]. Machine learning revolves around the creation of new patterns and the management of these patterns through algorithms. It can offer real-time detection of active threats, thereby aiding cybersecurity teams in proactively preventing security breaches [117]. Machine learning has a substantial impact on cybersecurity, as it facilitates a range of techniques for the detection and mitigation of cyber threats [118]. This section discusses the most common machine learning approaches used in strengthening the cybersecurity for industrial control systems.

### Anomaly Detection

This approach entails training a machine learning model on a dataset that represents normal behavior, enabling it to identify deviations or anomalies effectively [119]. It can help detect abnormal activities, such as network intrusions, system misuse, or suspicious user behavior [120]. The correct detection of unusual events empowers the decision maker to act on the system to correctly avoid, correct, or react to the associated situations [121].

The strength of different machine learning anomaly detection techniques can vary depending on the specific use case, dataset, and goals of the anomaly detection task. Table 3 shows some common machine learning anomaly detection techniques and their strengths.

The above mentioned machine learning techniques for anomaly detection are powerful tools for identifying outliers and unusual patterns in data, but they come with limitations and challenges. Table 4 highlights some common limitations of different machine learning anomaly detection techniques.

(a) Supervised machine learning-based anomaly detection approaches: Supervised machine learning-based anomaly detection approaches can be applied to strengthen the cybersecurity of ICS [140]. It involves training a model on labeled data to classify normal and abnormal behavior in the system. Here are a few common approaches [141]:
  - Support Vector Machines (SVM): SVM is a widely employed supervised learning algorithm for tasks involving classification [142]. In anomaly detection, SVM can be trained on labeled data, where normal behavior is labeled as one class and anomalies as another [143]. SVM tries to find a hyperplane that maximally separates the two classes. According to researchers, data in this domain is referred to as industrial sensor data because it is recorded using different sensors and collected for analysis. It has a temporal aspect, and time series analysis is also used in works such as Ref. [144], according to research conducted on the Petroleum industry, which is one of such real-world application scenarios. In particular, heavy extraction machines for pumping and generation operations such as turbo-machines are intensively monitored by hundreds of sensors each that send measurements with a high frequency for damage prevention. To deal with this and with the lack of labeled data for training and validation of models in some scenarios [145,146], an approach describing a combination of a fast and high-quality segmentation algorithm with a one-class support vector machine for efficient anomaly detection in turbomachines was suggested. Another researcher employed a technique that merges unsupervised fuzzy C-means clustering (FCM) with a supervised support vector machine (SVM) to compute the distance between communication data within industrial control networks and the cluster center. The support vector machine then categorizes data segments that meet specific threshold criteria. Experimental findings demonstrate that, in comparison to the conventional intrusion detection approach, this method can notably decrease the training duration and enhance classification accuracy, all without prior knowledge of class labels [147];
  - Random Forest: Random Forest is an ensemble learning technique that amalgamates numerous decision trees. In anomaly detection, each decision tree is trained on labeled data, and the final decision is made based on the majority vote of the trees. Random Forest can handle high-dimensional data and is effective at identifying anomalies. Random Forests are collections of Decision Trees, binary classifiers consisting of one root node, several internal split nodes, and leaf nodes that are used to classify events [148];
  - Neural Networks: Neural networks, such as feed-forward networks or recurrent neural networks (RNNs), can also be used for supervised anomaly detection. These models are trained using labeled data and have the capability to grasp intricate patterns and relationships within the data [149].
    RNNs are useful with ICS data. The employment of parallel multi-view neural networks to identify anomalies within an industrial control system has been studied recently. These networks achieve this by forecasting operational states. Integrating this predictive ability into the system enables semi-supervised monitoring of system operations, ensuring that the real-time system state aligns with a predefined region within the state space forecast earlier by the neural networks. Additionally, in cases where the two predictive models disagree in their assessment of the system’s state (leading to a lack of consensus), it is probable that the system’s operation has been compromised. This divergence could be attributed to issues such as faulty equipment, communication errors, or other sources of malfunction. To obtain distinct perspectives on the system, one of the predictive models is trained to analyze the data flow from system control packets, while the other model is trained to examine gyrometric signals collected from physical sensors within the control system [150];
  - Gradient Boosting: Gradient Boosting is an ensemble learning approach that blends several weak learners, such as decision trees, to construct a robust predictive model [151]. It iteratively builds models, focusing on the instances that previous models misclassified. Gradient Boosting algorithms like XGBoost or LightGBM have been successful in various anomaly detection tasks [152,153,154]. While the majority of machine learning methods concentrate on optimizing hyperparameters to enhance detection rates, alternative research suggests an approach that prioritizes the identification of the most promising dataset features. This approach employs Gradient Boosting Feature Selection (GBFS) to select these features before implementing the classification algorithm. This combination enhances not just the detection rate but also accelerates execution speed. GBFS employs the Weighted Feature Importance (WFI) extraction method to simplify classifier complexity. After identifying the most promising features from the power grid dataset using a GBFS module, it accesses a range of machine learning techniques based on decision trees [155,156];
  - Deep Learning Autoencoders: Autoencoders are neural network structures that acquire the ability to encode input data into a reduced-dimensional representation and subsequently decode it to reconstruct the original input. By training an autoencoder on a large dataset of normal behavior, it learns to reconstruct the normal data accurately [157]. Anomalies can then be detected by measuring the reconstruction error, where higher errors indicate anomalies. Deep Learning has demonstrated remarkable efficacy in autonomously acquiring valuable representations of intricate data [158]. Autoencoders excel at identifying the most challenging and nonlinear dependencies within the data, making them particularly adept at achieving high-quality anomaly detection [159]. In certain scenarios, autoencoders demand fewer computing resources. Some researchers have devised an intrusion detection system for recognizing various injection attacks, employing deep learning algorithms like stacked autoencoders and deep belief networks that are customized for this purpose [160]. A group of researchers endeavored to create an intrusion detection system reliant on deep learning. This system can swiftly detect intrusions and other undesirable activities that may disrupt networking systems. It leverages the One Hot encoder for preprocessing and the Autoencoder for feature extraction [161].
(b) Unsupervised machine learning-based anomaly detection approaches: These play a crucial role in enhancing the cybersecurity of ICS by identifying abnormal behavior or potential cyber threats without needing labeled data. Here are some common unsupervised anomaly detection approaches used in ICS:
  - Isolation Forest: Isolation Forest is a tree-based algorithm that isolates anomalies by recursively partitioning data until each data point is isolated in its tree leaf. Anomalies are identified as instances that require fewer partitions to isolate. The algorithm can be categorized into two main stages: Forest construction and element evaluation. During the creation of individual trees, the sample element set is divided. More precisely, nodes for isolating trees are created by randomly selecting an attribute and its associated partition. Conversely, the evaluation function focuses on navigating the analyzed element through these trees [162];
  - Density-Based Clustering (like DBSCAN): Density-based clustering methods group data points based on their density. In ICS, these methods can help identify clusters of normal behavior and consider isolated points as anomalies [119]. This approach is based on the assumption that data from healthy states tend to cluster in high-density regions, while data from faulty states are typically found in low-density regions. By delineating the boundaries of these regions, it becomes possible to identify data points corresponding to anomalous states. The method involves assessing the density values for both healthy and faulty machinery. The rate at which the density changes from healthy to faulty is determined as a fault threshold. This method can be particularly useful in scenarios where obtaining faulty data is arduous or expensive [163];
  - Local Outlier Factor (LOF): LOF calculates the density of data points with respect to their neighbors. In ICS, LOF can identify points with significantly lower density as anomalies [164]. LOF aims to forecast the imminent faults of an appliance in the IoT system, whose predictive performance greatly depends on the selection of its hyperparameters. Hyperparameter tuning for unsupervised machine learning models such as LOF in IoT systems presents a significant challenge due to the potential existence of previously unseen anomalies in incoming data, which were not part of the training set. A novel heuristic approach for hyperparameter tuning in LOF explicitly accounts for the likelihood of encountering new types of anomalies has been studied. Utilizing this novel approach to tune the LOF model resulted in robust predictive performance, as demonstrated in both simulation experiments and real-world data applications [165];
  - One-Class Support Vector Machine (OCSVM): One-Class SVM is designed for novelty detection and can be used in ICS to learn the boundaries of normal behavior and classify instances. OCSVM can train anomaly detection model with only one class of samples. Furthermore, OCSVM can build a more accurate model and has robustness for noise samples. OCSVM has been proven to be an effective machine learning method for intrusion detection in industrial control systems [166];
  - Gaussian Mixture Models: GMM assumes that data points are generated from a mixture of several Gaussian distributions. Anomalies can be detected as instances with low probabilities under the fitted GMM. In their study, certain researchers employed a statistical traffic analysis approach that relies on the Gaussian mixture model. This method was utilized to discern the presence of anomalies, such as man-in-the-middle attacks, within a communication process by analyzing the timing of traffic communication. In modern network environments, the usual communication process tends to demonstrate a significant level of stability during normal conditions, resulting in the convergence of its communication timings to a relatively consistent range of values. Nevertheless, if there is a departure in the time elapsed for traffic generated by a particular communication compared to the pattern observed in historical data, it is a reasonable indication that anomalies, like potential man-in-the-middle attacks, might be occurring in this process [167].
(c) Intrusion Detection Systems (IDS): Machine learning, when integrated into IDSs, has yielded favorable outcomes by leveraging various learning approaches, encompassing supervised, unsupervised, and reinforcement learning [168,169]. Machine Learning as a part of IDSs has had positive results by using different kinds of learning, including supervised, unsupervised, and reinforcement learning [170].
  - Random Forest (RF): In response to these challenges, a power industrial control system intrusion detection model based on Random Forest was introduced. Additionally, the same study introduced an enhanced grid search algorithm (IGSA) designed to optimize the hyperparameters of the RF intrusion detection model, thus enhancing its efficiency and effectiveness. The proposed IGSA significantly accelerates computation speed, reducing it from O(nm) to O(n × m). Following the hyperparameter optimization process, the suggested model was evaluated using a publicly available power industrial control system dataset. The experimental findings illustrate that our approach attains outstanding detection performance, achieving an impressive accuracy rate of 98%. Moreover, it surpasses comparable efforts within the same category [171]. The ensemble Gradient Boosting algorithm is an ensemble learning method based on a combination of additive models (weak learners), which can gradually learn from the previous misclassifications to create a stronger learning model [172]. This algorithm has been enhanced with a feature selection process, which elevates its overall performance by extracting the most pertinent features from the input data;
  - Unsupervised Learning: Unsupervised learning does not necessitate labeled data and proves beneficial when obtaining labeled data is scarce or challenging. Common unsupervised learning algorithms for IDS in ICS include:
    – Autoencoders: Autoencoders are neural network architectures that learn to reconstruct the input data. They can be used for anomaly detection by identifying instances with high reconstruction error [173]. These autoencoder-based methods have been applied to build NIDS in IoT environments recently. Some researchers used deep autoencoders to detect IoT botnet attacks. Their proposed model comprised an ensemble of autoencoders, with each autoencoder trained to recognize the normal network behaviors of a specific IoT device and flag any unusual traffic stemming from that device. This model was assessed on a testbed network containing nine commercial IoT devices that had been compromised by the Mirai and BASHLITE botnets. The model showcased exceptional performance, achieving a true positive rate of 100% while keeping the false positive rate at just 7% [174];
    – Isolation Forest: The Isolation Forest algorithm is rooted in the Decision Tree algorithm. It identifies outliers by randomly selecting a feature from the available feature set and then randomly choosing a split value within the range of that feature’s maximum and minimum values. This random partitioning of features leads to shorter paths in trees for anomalous data points, effectively distinguishing them from the majority of the data [175];
    – Density-Based Clustering: The hypothesis is that if two packets belong to the same attack type, they are more likely to fall into the same cluster when any clustering algorithm is applied with any hyperparameters. In other words, when several clustering algorithms are applied, the more two samples fall into the same cluster, the more likely they belong to the same attack type [176].
  - Semi-Supervised Learning: Semi-supervised Learning combines labeled and unlabeled data during training. This can be useful in scenarios where obtaining labeled data is costly or time-consuming:
    – Reinforcement Learning: Reinforcement learning can be used in IDS for dynamic decision-making in response to evolving cyber threats. However, it might be less commonly used due to the need for careful tuning and potential risks in real-world environments [177]. A team of researchers introduced an innovative approach to network intrusion detection, merging Q-learning-based reinforcement learning with a deep feed-forward neural network technique for the purpose of network intrusion detection [178]. The proposed Deep Q-Learning (DQL) model offers a persistent self-learning ability within a network environment. It employs an automated trial-and-error approach to identify various types of network intrusions and consistently improve its detection capabilities;
    – Deep Learning: Deep learning models, including recurrent neural networks (RNNs) and convolutional neural networks (CNNs), can be utilized in IDS to capture intricate patterns and temporal dependencies within ICS data [179].
  - Secure Authentication and Authorization: Machine learning can enhance authentication and authorization processes, making it more difficult for attackers to bypass access controls. Reinforcement learning methods, like Q-learning, can be utilized for IoT device authentication and the identification of jamming and malware attacks. These techniques acquire knowledge from the environment, whether on the cloud or high-computational edge devices, without relying on a pre-established training dataset [180];
  - Leveraging Machine Learning for Predictive Maintenance in ICS: A group of researchers introduced a Predictive Maintenance approach that enables the adoption of dynamic decision rules for maintenance management, even in scenarios involving high-dimensional and censored data challenges. This is accomplished by training multiple classification modules with varying prediction horizons, offering diverse performance trade-offs concerning the frequency of unforeseen failures and unused equipment lifespan. Subsequently, this information is integrated into an operational cost-driven maintenance decision system to minimize anticipated expenses. The efficacy of this methodology is showcased through both a simulated illustration and a benchmark maintenance problem in semiconductor manufacturing [181].

## 9. Challenges of Machine Learning Approaches and Mechanisms for Defending ICS

ML methods have demonstrated significant potential in improving cybersecurity but encounter various challenges. Some of the key challenges of using machine learning in cybersecurity include:

- Data Quality and Quantity: ML models require large amounts of high-quality, labeled data for training. In cybersecurity, obtaining such data can be difficult due to the scarcity of certain types of cyber attack data or the potential risks associated with using real-world attack data [182]. Another challenge in ML-based systems is the dependency on data labeling. Large datasets with labeled data are necessary for ML-based systems, which are challenging and expensive to gather [183];
- Imbalanced Data: In cybersecurity, the number of normal instances (benign data) often outweighs the number of malicious instances (attack data), resulting in imbalanced datasets. This can lead to biased models and poorer performance in detecting rare cyber threats [184];
- Adversarial Attacks: Adversaries can attempt to manipulate ML models by crafting adversarial examples, which are carefully designed inputs to cause misclassification. Adversarial attacks can reduce the reliability and robustness of ML-based cybersecurity solutions [185]. As per findings from certain researchers, it is possible for an adversary to target the training process directly. If the adversary manages to introduce their own data samples or manipulate the training data in some way, they can effectively manipulate the model, leading to erroneous associations between input characteristics and categories (referred to as “false learning”) or undermining the trustworthiness of the labeling, ultimately resulting in a reduction in the accuracy of the model. In both scenarios, tampering with the training process undermines the model’s reliability and weakens its ability to withstand adversarial inputs;
- Logic manipulation: In this instance, a malicious actor targets the machine learning model by manipulating the model’s logic in order to interfere with the learning outcomes. This is regarded as one of the most significant threats to the machine learning process [186]. A single machine learning model may not be universally suitable for all tasks across different scenarios that require attention. Typically, a specific machine learning model is trained for a particular problem or, at best, can be adapted for a similar task. Furthermore, Cyber-Physical Systems (CPS) exhibit considerable diversity, making it challenging to apply a single machine learning model comprehensively. Therefore, a range of models and diverse datasets are essential to create system-wide solutions [187];
- Machine learning models impose stringent demands regarding the dimensions, configurations, and formats of input data: Despite the vast amounts of data that Cyber-Physical Systems (CPS) collect, there is no assurance of data quality, particularly as the lifespan of newly introduced IoT hardware may remain unverified. To prepare input data for machine learning, it must undergo transformation from its raw state into a specific data format. This transformation process can result in substantial computational expenses. Alternatively, machine learning systems must possess the capability to inherently manage and adapt to the raw data and associated noise.

## 10. Recommendations and Future Research Direction

- Threat Modeling and Risk Assessments: Conduct thorough threat modeling and risk assessments to identify critical assets, vulnerabilities, and potential attack vectors. Use this information to prioritize security measures based on risk levels [188]. Since attackers are able to monitor non-encrypted traffic to gain information about the system, machine learning approaches that can integrate threat modeling, risk assessment, and automatic traffic encryption will be a great resource for strengthening the cyber defense of industrial control systems;
- Security-by-Design: Incorporate cybersecurity considerations from the early stages of ICS development and implementation. Security-by-design principles can help minimize vulnerabilities and reduce the cost of retroactively implementing security measures;
- Remote Continuous Monitoring and Incident Response: Implement real-time monitoring of ICS networks and establish efficient incident response plans to quickly detect and mitigate cyber threats with less human intervention;
- Intelligent Hardware Security: Develop and adopt hardware security measures, such as secure boot, cryptographic processors, and physical tamper detection, to enhance the overall security posture of ICS components;
- Develop diverse machine learning models: Machine learning models should be trained to manage diverse tasks to make it possible to address more than one security situation. Machine learning models trained for a specific problem, or can at most be retrained to another similar task;
- Automation and Response: Artificial Intelligence can automate the response to certain security incidents. For example, AI-driven security orchestration can isolate compromised systems, block malicious traffic, and initiate incident response processes;
- Collaborative Threat Intelligence: Artificial Intelligence can facilitate the sharing of threat intelligence among organizations, enabling them to collectively defend against emerging threats that target ICS environments;
- Robustness Testing and Simulation: AI can assist in simulating potential attack scenarios to identify vulnerabilities and weaknesses in ICS systems, helping organizations proactively strengthen their security posture;
- Continuous Monitoring and Updating: Cyber threats evolve, so it is crucial to continuously monitor the system’s performance and update the machine learning models accordingly. Regularly retraining the models with new data and adapting to emerging attack patterns is necessary.

## 11. Conclusions

This article has comprehensively examined the intricate facets of ICS security. Its primary goal has been to provide novel insights and foster the growth of knowledge within the ICS security domain. Throughout the article, considerable attention has been dedicated to the exploration of the fundamental elements influencing decision-making in ICS design. A broad spectrum of established security measures has been meticulously evaluated, alongside an in-depth analysis of the integration of cutting-edge methodologies such as machine learning to fortify security measures. The holistic approach taken within the article ensures a profound comprehension of the complexities inherent to ICS security, thereby proposing the integration of machine learning, particularly in the context of training models for diverse tasks, as a potential solution to address a myriad of security scenarios. Additionally, it analyses the necessity of incorporating cybersecurity considerations right from the outset of ICS development. Regular retraining of machine learning models with up-to-date data to effectively adapt to evolving attack patterns has been recommended in this article. The article’s emphasis on both the identification of challenges and the formulation of practical recommendations not only communicates the current findings but also lays a solid foundation for future research endeavors and improvements in the realm of ICS security.

The insights presented in this article serve to advance the field of ICS security and provide valuable guidance for enhancing the security of critical infrastructure. By considering the recommended strategies we shall continue the research and support organizations to better protect their ICS environments against evolving cyber threats and contribute to the overall resilience and reliability of Industrial systems.

Furthermore, it is essential for future research to maintain a dynamic approach to ICS security. The landscape of threats is in a constant state of flux, with adversaries continuously refining their tactics. Consequently, upcoming investigations should prioritize the development of adaptable security strategies capable of responding to emerging threats. This entails the establishment of self-learning security systems with the ability to dynamically adjust their defensive mechanisms, making effective use of artificial intelligence and machine learning for real-time threat intelligence and preemptive threat mitigation. Additionally, fostering interdisciplinary cooperation between experts in cybersecurity, control systems engineering, and data science is crucial for crafting holistic security solutions that encompass both the technical and operational dimensions. In an era marked by the proliferation of digitalization and increased connectivity in industrial contexts, proactively outmaneuvering adversaries and preemptively countering nascent risks holds paramount importance in preserving the integrity of critical infrastructure and upholding the dependability of industrial systems.

## Figures and Tables

**Table 1:** Potential ICS threat event.

Threat	Description
Denial of control action	Control systems can face disruptions when the flow of information is deliberately delayed or blocked, resulting in the unavailability of networks to control system operators. This can manifest as bottlenecks in information transfer or as a denial of service, particularly when related to IT-resident services such as DNS
Unauthorized reprogramming of control devices	Unauthorized modifications to programmed instructions in PLCs, RTUs, DCS, or SCADA controllers, along with alterations to alarm thresholds or unauthorized commands issued to control equipment, have the potential to lead to various adverse outcomes. These consequences may include equipment damage if operational tolerances are exceeded, premature shutdown of processes (such as the untimely shutdown of transmission lines), triggering environmental incidents, or even the disabling of control equipment
Spoofed System Status Information	The transmission of false information to control system operators can serve two main purposes: to conceal unauthorized changes or to instigate improper actions by system operators
Control Logic Manipulation	Control system software or configuration settings modified, producing unpredictable results
Safety Systems Modified	Safety systems operation are manipulated so that they either (1) do not operate when needed or (2) perform incorrect control actions that damage the ICS
Malware on Control Systems	Malicious software (e.g., virus, worm, Trojan horse) introduced into the system

**Table 2:** Vulnerabilities of the proposed protocols.

Protocol	Lack of Integrity	Lack of Confidentiality	Lack of Availability	Lack of Authentication	Lack of Authorization	Lack of Encryption	Reference
DNP3	✓			✓	✓	✓	[85]
Modbus	✓		✓	✓			[86]
IEC 60870-5-104	✓	✓	✓	✓			[87]
IEC 61850				✓		✓	[88]
IEC 61400-25	✓						[89]
IEEE C37.118	✓	✓	✓				[90]

**Table 3:** Strength of different machine learning techniques for anomaly detection.

Technique	Effective in High-Dimensional Spaces	Non-Linearity Handling	Tunable Margin	Robust to Noisy Data	Feature Importance	Scalability	Ease of Interpretation	Feature Learning	References
Support Vector Machines	✓	✓	✓						[122]
Random Forest	✓			✓	✓	✓			[123]
Neural Networks	✓	✓				✓		✓	[124]
Gradient Boosting		✓			✓	✓			[125]
Autoencoders			✓	✓				✓	[126]
Isolation Forest	✓	✓				✓	✓		[127]
Density-based Clustering	✓			✓				✓	[128]
Local Outlier Factor	✓			✓			✓		[129]
Gaussian Mixture Models						✓	✓		[130]

**Table 4:** Limitations of different machine learning techniques for anomaly detection.

Technique	Computationally Expensive	Not Scalable	Lack of Interpretability	Limited Multiclass Anomaly Detection	Lack of Interpretability	Difficulty with Multimodal Data	Limited for Time-Series Data	Difficulty with Highly Imbalanced Data	References
Support Vector Machines	✓		✓	✓	✓			✓	[131]
Random Forest	✓				✓	✓	✓	✓	[132]
Neural Networks	✓							✓	[133]
Gradient Boosting	✓		✓	✓					[134]
Autoencoders					✓			✓	[135]
Isolation Forest						✓	✓	✓	[136]
Density-based Clustering		✓		✓	✓	✓			[137]
Local Outlier Factor	✓	✓		✓	✓				[138]
Gaussian Mixture Models		✓			✓	✓		✓	[139]

## Data Availability

Not applicable.

## Abbreviations

The following abbreviations are used in this manuscript:

IOT	Internet of Things
ICS	Industrial Control System
CPS	Cyber Physical System
DCS	Distributed Control system
SCADA	Supervisory Control and Data Acquisition
PLC	Programmable Logic Controllers
RTU	Remote Telemetry Units
WAN	Wide Area Networks
HMI	Human-machine interface
IDS	Intrusion Detection System
IED	Electromagnetic Device
DNP3	Distributed Network Protocol 3
OS	Operating System
LAN	Local Area Network
EMP	Electromagnetic Pulse
ASDU	Application Service Data Unit
MMQTT	Message Queuing Telemetry Transport
OPC	Open Platform Communications
